# Gut microbial change after administration of *Lacticaseibacillus paracasei* AO356 is associated with anti-obesity in a mouse model

**DOI:** 10.3389/fendo.2023.1224636

**Published:** 2023-08-29

**Authors:** Eun-Ji Song, Eun-Sook Lee, Young In Kim, Dong-Uk Shin, Ji-Eun Eom, Hee Soon Shin, So-Young Lee, Young-Do Nam

**Affiliations:** ^1^ Food Functionality Research Division, Korea Food Research Institute, Wanju-gun, Republic of Korea; ^2^ Department of Pharmacology, Asan Medical Center, University of Ulsan College of Medicine, Seoul, Republic of Korea; ^3^ Bio-medical Institute of Technology, University of Ulsan College of Medicine, Seoul, Republic of Korea; ^4^ Department of Food Biotechnology, Korea University of Science and Technology, Daejeon, Republic of Korea

**Keywords:** lacticaseibacillus paracasei, lab, probiotics, gut microbiota, obesity

## Abstract

**Introduction:**

The status of an impaired gut microbial community, known as dysbiosis, is associated with metabolic diseases such as obesity and insulin resistance. The use of probiotics has been considered an effective approach for the treatment and prevention of obesity and related gut microbial dysbiosis. The anti-obesity effect of *Lacticaseibacillus paracasei* AO356 was recently reported. However, the effect of *L. paracasei* AO356 on the gut microbiota has not yet been identified. This study aimed to elucidate the effect of *L. paracasei* AO356 on gut microbiota and ensure its safety for use as a probiotic.

**Methods:**

Oral administration of *L. paracasei* AO356 (10^7^ colony-forming units [CFU]/mg per day, 5 days a week, for 10 weeks) to mice fed a high-fat diet significantly suppressed weight gain and fat mass. We investigated the composition of gut microbiota and explored its association with obesity-related markers.

**Results:**

Oral administration of *L. paracasei* AO356 significantly changed the gut microbiota and modified the relative abundance of *Lactobacillus*, *Bacteroides, and Oscillospira*. *Bacteroides and Oscillospira* were significantly related to the lipid metabolism pathway and obesity-related markers. We also confirmed the safety of *L. paracasei* AO356 using antibiotics resistance, hemolysis activity, bile salt hydrolase activity, lactate production, and toxicity tests following the safety assessment guidelines of the Ministry of Food and Drug Safety (MFDS).

**Discussion:**

This study demonstrated that *L. paracasei* AO356 is not only associated with an anti-obesity effect but also with changes in the gut microbiota and metabolic pathways related to obesity. Furthermore, the overall safety assessment seen in this study could increase the potential use of new probiotic materials with anti-obesity effects.

## 1 Introduction

Obesity is a global public health concern associated with a wide range of health problems, including diabetes, cardiovascular disease, and cancer. Obesity, characterized by an excessive accumulation of body fat, is a complex and multifactorial condition resulting from a combination of factors such as genetics, environment, and behavior (1). While traditional interventions such as diet and exercise are effective in obesity management, emerging evidence suggests that gut microbiota may also play a role in regulating body weight and inflammation related to obesity (2, 3). Gut microbiota influences nutrient absorption, energy balance, and the immunologic system, which contribute to the modulation of body homeostasis and metabolism (4–6).

Probiotics are microorganisms that confer a health benefit on the host when administered in adequate amounts (7). Probiotics are thought to exert their beneficial effects through various mechanisms, including modulating the gut microbiota, strengthening the gut barrier, and interacting with the host immune system (8). Therefore, the use of probiotics may serve as an effective approach to the treatment and prevention of obesity via modulation of the gut microbiota. Our previous study suggested that *Lacticaseibacillus paracasei* AO356 exerts its anti-obesity effect by regulating adipogenesis and thermogenesis (9). However, the effect of *L. paracasei* AO356 on gut microbiota was not confirmed. Lactic acid bacteria including *Lactobacillus* have been reported to have functions related to the inhibition of pathogen colonization, cholesterol metabolism, liver function, and fat accumulation (10–14). Moreover, the anti-obesity effect of *L. paracasei* strains are reported in several animal and human studies (15–17). Of note, *L. paracasei* K56 exerts its anti-obesity effect by modulating the gut microbiota in high-fat diet (HFD)-induced obesity mice (18). Therefore, we expect the changes in the gut microbiota to be related to the anti-obesity effect of *L. paracasei* AO356.

As many beneficial effects of probiotics have been reported, safety concerns such as antibiotic resistance and toxicity of strains have been raised as important issues for probiotics selection. In 2021, the Korea Ministry of Food and Drug Safety (MFDS) presented guidelines regarding the safety of probiotics (19). Thus, in this study, we investigated the effect of *L.paracasei* AO356 on gut microbiota and its association with obesity and evaluated its safety following the guidelines presented by MFDS. Our findings suggest that *L.paracasei* AO356 may be a promising strategy for the management of obesity and its associated health problems.

## 2 Materials and methods

### 2.1 Preparation for L. paracasei AO356

L. paracasei AO356 (KCCM12145P), isolated from fecal samples of healthy Koreans in general dietary pattern, was cultured in the MRS broth medium (BD Difco co. USA) at 37°C for 24 h aerobically, collected by centrifugation (1000 × g for 20 min), and washed twice with sterilized PBS. Freeze-dried cells were then stored at -80°C until they were administered to the mice. The colony forming unit (CFU) with freeze-dried cells was determined before they were used.

### 2.2 An animal experiment

C57BL/6 mice (thirty 6-week-old males) purchased from Central Lab Animal, Inc. (Seoul, Korea) were housed under standard laboratory conditions (22 ± 2°C, 12 h light/dark cycle) and acclimatized to the lab environment for 1 week. The animals were randomly allocated into three groups. One group was fed a normal chow diet (ND, Teklad 2018S; Envigo Research Indianapolis, Indiana, USA). The other two groups were fed a high-fat diet (HFD) comprising 55% fats (D12492 formula of Research Diet, Saeron Bio, Gyeonggi, Korea) with or without *L. paracasei* AO356. The diets were provided *ad libitum*, and *L. paracasei* AO356 was administered at a dose of 5 × 10^7^ CFU/mg in 100 µL of PBS by oral gavage 5 days a week for 10 weeks. Two mice were housed per cage and their body weights were measured once a week (n=9 for ND, n=8 for HFD and n=9 for HFD + AO356). At the end of the experiment, fresh feces were collected and stored at −80°C for gut microbiome analysis. All animals were then subjected to overnight fasting and sacrificed. Tissues, including epididymal white fat tissue (EFT), retroperitoneal white fat tissue (RFT), inguinal white fat tissue (IFT), and intrascapular brown adipose tissue (BAT), were immediately excised and weighed. The concentrations of glucose, triglycerides, LDL cholesterol, and HDL cholesterol in serum were determined by appropriate enzyme kinetic or colorimetric assays (Roche, Mannheim, Germany) on a Hitachi automatic analyzer Modular Analytics (Hitachi, Japan). Serum insulin was quantified using ELISA assay kits (ALPCO, Diagnostics, NH, USA). Homeostasis model assessment for insulin resistance (HOMA-IR) was calculated via the following equation (20): fasting glucose (mg/dL) × fasting insulin (µU/mL) ÷ 2430. This experiment was approved by the Korea Food Research Institutional Animal Care and Use Committee (approval # KFRI-M-16047) following their guidelines and regulations on the care and use of laboratory animals.

### 2.3 Gut microbiota analysis

Metagenomic DNA from feces was extracted using the QIAamp DNA Stool Mini Kit (Qiagen) with an additional bead-beating step as previously described (21). The V1-V2 hypervariable region of the bacterial 16S rRNA genes was amplified by PCR using universal primers (8F and 338R) with barcode sequences for multiplexing reads of each sample. Amplicon sequencing was performed on an Ion Torrent PGM system (Thermo Scientific, DE, USA) with a 318D sequencing chip. Raw sequence reads were quality-filtered and quality-controlled reads were processed for diversity analysis and taxonomy assignment using the Quantitative Insights into Microbial Ecology 1 (QIIME1) software package (22). The molecular functions of each sample were predicted based on 16S rRNA data in the Phylogenetic Investigation of Communities by Reconstruction of Unobserved States (PICRUSt) software (23).

### 2.4 Determination of minimum inhibitory concentration (MIC) of antibiotics

The MIC of AO356 was determined with an ETEST® strip (BioMérieux, SA, France). AO356 was diluted in 0.9% saline (0.5~1.0 McFarland standard) and streaked on MRS agar plates. After the plates were dried, ETEST® strips for ampicillin (AM), chloramphenicol (CL), clindamycin (CM), erythromycin (EM), gentamicin (GM), kanamycin (KM), streptomycin (SM), tetracycline (TC), and vancomycin (VA) were applied to the plates. The antibiotics concentration scale of the strips was from 0.016 μg/mL to 256 μg/mL. The agar plates were incubated anaerobically at 37°C for 48 h.

### 2.5 Hemolysis activity test

Hemolysis activity was evaluated by the plate assay described by the Ministry of Food and Drug Safety (MFDS) for microbiology guidelines. A loop of AO356 was streaked on a sheep blood agar plate and incubated at 37°C for 24 h. *Escherichia coli* (ACTC 1682) was cultured and used as a positive control. Hemolysis activity was determined by the form of hemolytic cells formed around colony on the plate (24, 25). Beta-hemolysis causes a clear zone with the transparency of the base medium surrounds the colony when observed under the light. Alpha hemolysis causes a green or brown discoloration in the medium. Gamma-hymolysis indicates the lack of hemolysis.

### 2.6 Bile salt hydrolase (BSH) activity test

The bile salt hydrolase activity was tested using MRS agar plates including 0.5% taurodeoxycholic acid (MRS-TDCA). AO356 was then streaked on MRS-TDCA and incubated anaerobically at 37°C for 48 h. The BSH activity was determined by the visible halo surrounding colonies (26).

### 2.7 Toxicity test with Lactate dehydrogenase (LDH) assay

The Caco-2 cell line (human epithelial colorectal adenocarcinoma cell line; ATCC HTB-37) was seeded into a 24-well plate at a density of 1×10^5^ cells/well with a minimum essential medium (MEM) containing 10% fetal bovine serum and 1% penicillin-streptomycin and incubated at 37°C and 5% CO_2_ for 2 days. AO356 was treated on a Caco-2 cell with 1×10^7^, 1×10^8^, and 1×10^9^ CFU/well and incubated at 37°C and 5% CO_2_ for 24 h. *Escherichia coli* (ACTC 1682) was used as a positive control. The test wa perfomed in triplicate. The supernatant was centrifuged at 5,000 g for 15 min and used as a sample. The toxicity of AO356 was determined using an LDH assay kit following the manufacturer’s protocol (ab65393, Abcam, Cambridge, UK). The non-treated culture medium was used as a low control, the cell lysis buffer was used as a high control, and *Escherichia coli* (ACTC1682), known to be toxic, was used as a positive control.

### 2.8 D-lactate production test

The D-lactate production was determined using a D-/L-lactic acid assay kit following the manufacturer’s protocol (K-DLATE, Megazyme Ltd., Wicklow, Ireland). AO356 was inoculated on MRS broth and incubated at 37°C for 24 h. The culture medium was centrifuged at 5,000 g for 15 min and the supernatant was used for measurement of lactate production. *Lactobacillus rhamnosus* GG was used as a positive control.

### 2.9 Statistical analysis

Statistical significance was evaluated using a one-way analysis of variance (ANOVA) followed by Tukey’s a *post-hoc* test or the Kruskal-Wallis test followed by Dunn’s test. Permutational multivariate analysis of variance (PERMANOVA) statistical analyses were conducted on the Bray-Curtis dissimilarity with 999 permutations using the adonis2 function in the vegan package in R (27). Statistical significance was set at p<0.05. To identify taxa with differing relative abundances among groups, linear discriminant analysis effect size (LEfSe) analyses were conducted using the web-based program Galaxy (28). The strength of relationships between parameters was assessed using the Spearman correlation test.

## 3 Results

### 3.1 Effects of *L. paracasei* AO356 on HFD-induced obesity mice

**Figure 1:**
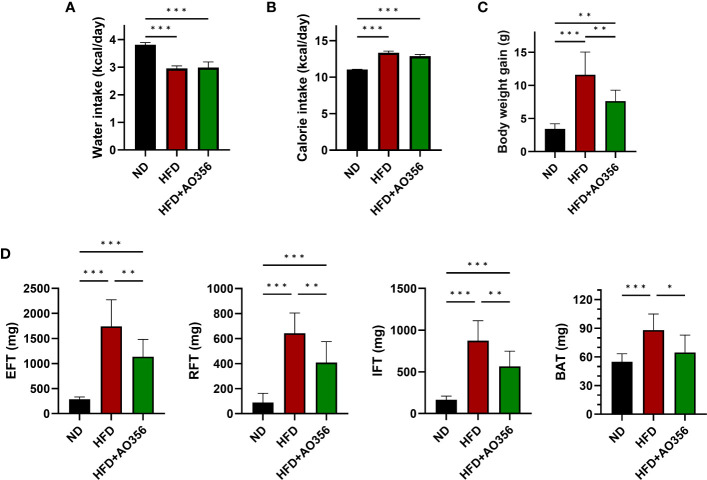
Effect of *L. paracasei* AO356 on weight and fat accumulation in a high-fat diet (HFD)-induced obese model after intervention for 10 weeks. **(A)** Water intake **(B)** Calorie intake **(C)** Body weight **(D)** Fat weight. Statistical significance was assessed using one-way ANOVA and Tukey’s multiple comparison test. The significance is indicated by *P < 0.05, **P < 0.01, ***P < 0.001. ND, normal diet mice; HFD, HFD mice; HFD+AO356, HFD mice with *Lacticaseibacillus paracasei* AO356 intervention; EFT, epididymal white fat tissue; RFT, retroperitoneal white fat tissue; IFT, inguinal white fat tissue; BAT, brown fat tissue.

To examine the effects of *L. paracasei* AO356 on obesity, we established an HFD-induced obesity mice model. Food and water intake was monitored every 2–3 days, and daily food intake and calories consumed were calculated. HFD feeding led to a significant decrease in water intake and an increase in calorie intake, body weight, and fat accumulation compared with the normal diet (NOR) group (**Figure 1**). There was no significant difference in water and calorie intake between the HFD group (HFD) and the HFD group treated with *L. paracasei* AO356 (HFD+AO356) (**Figures 1A, B**). Notably, 10 weeks of *L. paracasei* AO356 intervention affected body weight and fat accumulation upon HFD feeding. *L. paracasei* AO356 intervention significantly decreased body weight by 34.21% compared with that in the HFD group (**Figure 1C**). Additionally, *L. paracasei* AO356 intervention significantly decreased the weights of EFT (34.83%), RFT (36.51%), IFT (35.40%) and BAT (26.56%) (**Figure 1D**).

The HFD led to significant increases in insulin resistance and serum lipid profiles (**Supplementary Figure 1**). *L. paracasei* AO356 intervention significantly decreased the levels of glucose (16.07%), insulin (36.60%), and HOMA-IR (15.82%) which were increased by the HFD. Although microbial intake improved blood markers that were increased by the HFD, only glucose levels showed a significant difference between the *L. paracasei* AO356 intervention group and the HFD group (**Supplementary Figures 1A-C**). *L. paracasei* AO356 intervention also significantly decreased the TG levels by 11.42%. In addition, HDL and LDL cholesterol were decreased by 5.81% and 21.69%, respectively, but this decrease was not statistically significant when comparing the *L. paracasei* AO356 intervention group to the HFD group (**Supplementary Figures 1D-F**). These results indicate that *L. paracasei* AO356 has anti-obesity effects such as preventing weight gain and fat accumulation.

### 3.2 Gut microbial change after administration of *L. paracasei* AO356

**Figure 2:**
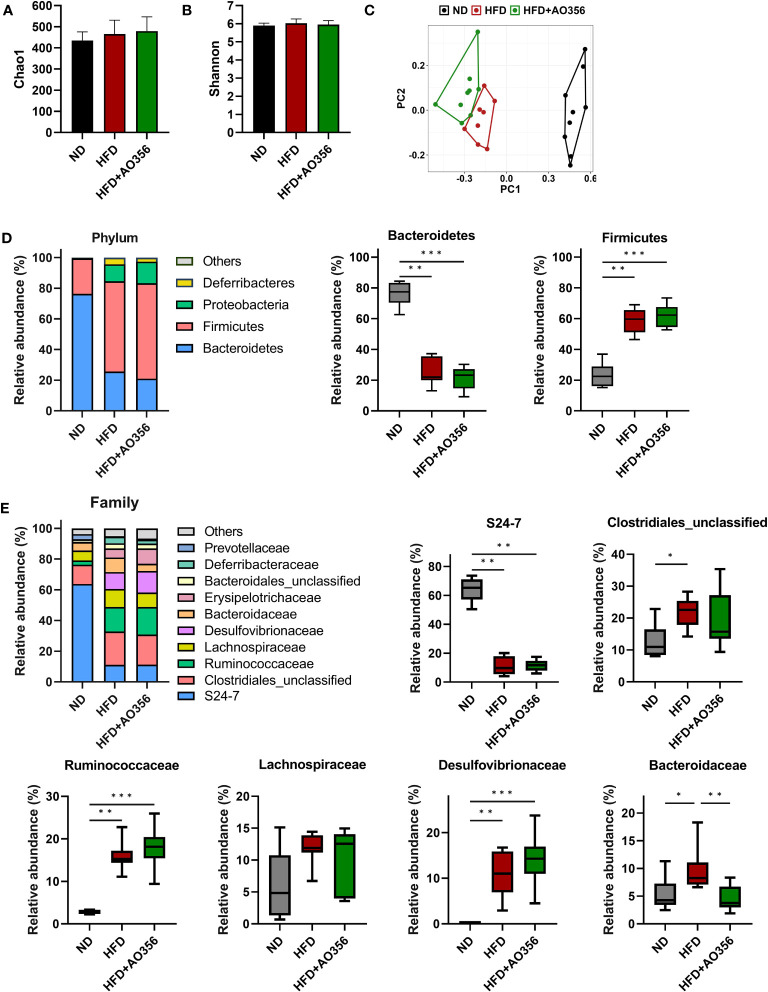
Effect of *Lacticaseibacillus paracasei* AO356 on the structure of gut microbiota in a high-fat diet (HFD)-induced obese model after intervention for 10 weeks. **(A)** Chao1 alpha diversity index. **(B)** Shannon alpha diversity index. **(C)** principal coordinate analysis (PCoA) plot based on Bray-Curtis dissimilarity. **(D)** Stacked bar chart of gut microbiota at phylum level and the relative abundance of phyla in each group. **(E)** Stacked bar chart of gut microbiota at phylum level and the relative abundance of families in each group. ND, normal diet mice; HFD, HFD mice; HFD+AO356, HFD mice with *Lacticaseibacillus paracasei* AO356 intervention; Data in the box plots are presented as the mean ± SD. Statistical significance was assessed using the Kruskal–Wallis test and Dunn’s multiple comparison test. The significance is indicated by *P < 0.05, **P < 0.01, ***P < 0.001.

The alpha diversity indices such as Chao1 and Shannon were similar among all groups (**Figures 2A, B**). However, the results of PCoA on the Bray-Curtis distance matrix showed differences among groups (**Figure 2C** **).** The ND group and the other two groups were clustered into two areas. The HFD and the *L. paracasei* AO356 intervention groups did not overlap and could be distinguished. The composition and the relative abundance of bacteria at the phylum level are shown in **Figure 2D**. In the ND group, the gut microbiota was dominated by *Bacteroidetes* followed by *Firmicutes*. Conversely, in the other two groups, *Firmicutes* was predominant, followed by *Bacteroidetes*. The HFD significantly decreased the relative abundance of *Bacteroidetes* and increased the relative abundance of *Firmicutes* in the HFD group compared to the ND groups. However, there was no significant difference in the relative abundance of *Bacteroidetes* and *Firmicutes* between the HFD and HFD+AO356 groups. **Figure 2E** presents the composition and relative abundance of the gut microbiota at the family level. In the ND group, the gut was dominated by the S24-7 family, followed by an unclassified family of *Clostridiales*. In contrast, in the HFD and HFD+AO356 groups, the S24-7 family, and unclassified families of *Clostridiales*, *Ruminococcaceae*, *Lachnospiraceae*, and *Desulfovibrionaceae* shared the predominance. Of note, the *L. paracasei* AO356 intervention significantly restored the relative abundance of *Bacteroidaceae*, which was significantly increased by the HFD when compared to the ND group.

### 3.3 Association of gut microbiota with body weight and fat accumulation

**Figure 3:**
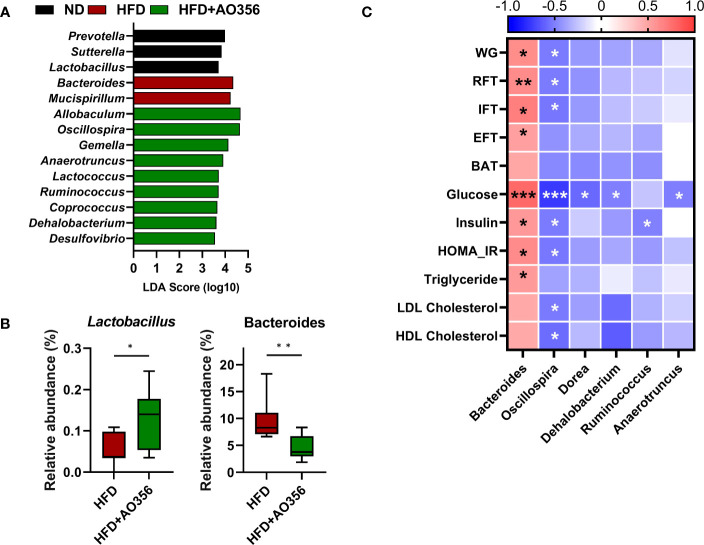
Association of microbiota *Lacticaseibacillus paracasei* AO356 and obesity-related markers. **(A)** LEfSe analysis identifies the significantly differently abundant taxa with a cutoff value of log10(LDA score) above 2.0. **(B)** Box plot of the relative abundance of Lactobacillus and Bacteroides. **(C)** A heatmap generated using Spearman correlation shows the association of genera with obesity-related markers. HFD, HFD mice; HFD+AO356, HFD mice with *Lacticaseibacillus paracasei* AO356 intervention; Data in the box plots are presented as the mean ± SD. Statistical significance was assessed using the Mann–Whitney test. The significance is indicated by *P < 0.05, **P < 0.01, ***P < 0.001. The color scale of the heatmap indicates the value of the correlation coefficient.

LEfSe analysis was performed to identify significantly differential genera among groups (**Figure 3A**). The ND group had a higher relative abundance of *Prevotella*, *Sutterella*, and *Lactobacillus*. The HFD group had a higher relative abundance of *Bacteroides* and *Mucispirillum*. The HFD+AO356 group had a higher relative abundance of nine genera including *Allobaculum*, *Oscillospira*, and *Ruminococcus*. Moreover, *L. paracasei* AO356 intervention significantly increased the relative abundance of *Lactobacillus* and decreased the relative abundance of *Bacteroides* in the HFD+AO356 group compared to the HFD group (**Figure 3B**). A Spearman correlation analysis was performed to identify the association of gut microbial genera with obesity-related markers (**Figure 3C**). Six genera were found to be significantly correlated with one or more obesity-related markers. In particular, *Bacteroides* showed a positive correlation with body weight, fat accumulation (RFT, IFT, and EFT), insulin resistance (Glucose, insulin, and HOMA-IR), and triglyceride levels. In contrast, *Oscillospira* showed a negative correlation with body weight, fat accumulation (RFT and IFT, insulin resistance (Glucose, insulin, and HOMA-IR), and lipid profiles (LDL and HDL cholesterol). These results suggested that the *L. paracasei* AO356 intervention-induced changes in gut microbiota are linked to its anti-obesity effects such as preventing weight gain and fat accumulation.

### 3.4 Functional metagenome prediction analysis

**Figure 4:**
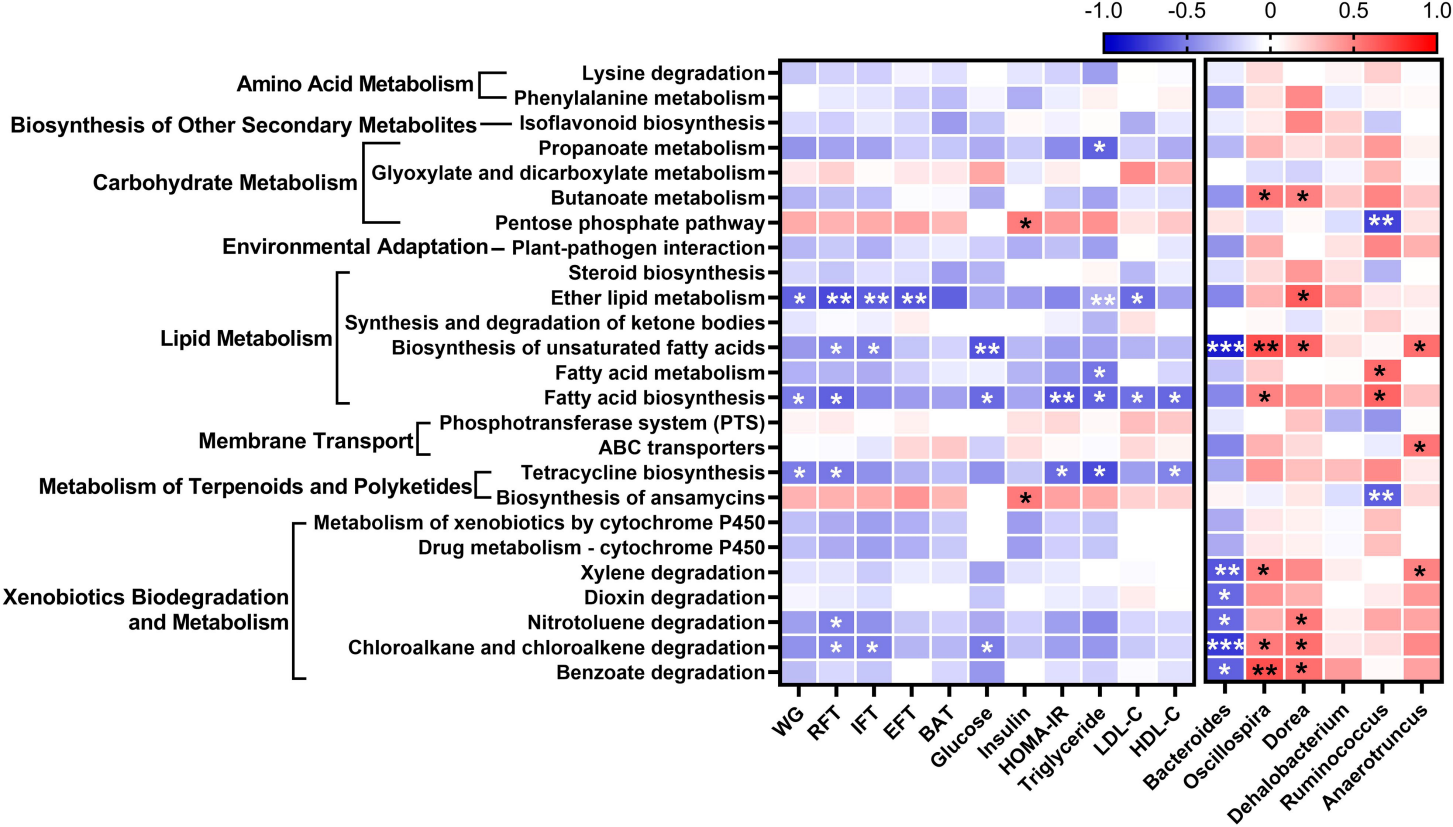
Association of predicted functional metagenome with gut microbiota and obesity-related markers. A heatmap generated using Spearman correlation shows the association of predicted functional metagenome at level 3 with obesity-related markers and genera that correlate with obesity-related markers. The significant correlations are indicated by *P < 0.05, **P < 0.01, ***P < 0.001. The color scale indicates the value of the correlation coefficient.

To evaluate differences in functional attributes of gut microbiota in response to *L. paracasei* AO356 intervention, predicted functional metagenomic profiles based on the Kyoto Encyclopedia of Genes and Genomes (KEGG) pathways were generated using PICRUSt. Twenty-five pathways were found to be associated with *L. paracasei* AO356 intervention (**Supplementary Table 1**). The *L. paracasei* AO356 intervention resulted in the enrichment of the biosynthesis of other secondary metabolites (isoflavonoid biosynthesis), carbohydrate metabolism-related pathways (butanoate metabolism, propanoate metabolism, and pentose phosphate metabolism), and lipid metabolism-related pathways (biosynthesis of unsaturated fatty acids, fatty acid metabolism, and ether lipid metabolism). Spearman’s correlation analysis was performed to identify the association of enriched pathways with obesity-related markers and genera (**Figure 4**). Ten pathways were found to be significantly correlated with one or more obesity-related markers. Of note, lipid metabolism-related pathways (Ether lipid metabolism, biosynthesis of unsaturated fatty acids, fatty acid biosynthesis) are strongly negatively correlated with obesity-related markers. In particular, biosynthesis of unsaturated fatty acids showed a negative correlation with RFT, IFT, glucose, and *Bacteroides*, and a positive correlation with *Oscillospira*, *Dorea*, and *Anaerotruncus*. Additionally, Fatty acid biosynthesis showed a negative correlation with body weight, RFT, HOMA-IR, TG, and HDL-C, and a positive correlation with *Oscillospira*. These results suggested that the *L. paracasei* AO356 intervention induced the modulation of lipid metabolism such as ether lipid metabolism, biosynthesis of unsaturated fatty acids, and fatty acid biosynthesis linked to the changes in gut microbiota such as *Bacteroides*, *Oscillospira*, and *Dorea*, resulting in an anti-obesity effect.

### 3.5 Safety evaluation

To evaluate the safety of *L. paracasei* AO356, we examined the antibiotics resistance, hemolysis activity, bile salt hydrolase (BSH) activity, measurements of D-/L-lactate production, and toxicity using an LDH assay which included safety assessment items presented in the food safety guidelines (29). The antibiotic resistance of *L. paracasei* AO356 for ampicillin, chloramphenicol, clindamycin, erythromycin, gentamicin, kanamycin, streptomycin, tetracycline, and vancomycin was determined using an E-test strip. The MIC values of *L. paracasei* AO356 were lower than the cut-off in the the European Safety Food Authority (ESFA) guidelines, suggesting that *L. paracasei* AO356 is susceptible to 9 antibiotics (**Supplementary Table 2**). In the hemolysis activity test, *L. paracasei* AO356 exhibited non-hemolytic activity (γ-hemolysis). Conversely, *Escherichia coli*, which is known to be toxic, showed clear hemolytic activity (β-hemolysis) (**Supplementary Figure 2A**). We examined the BSH activity of *L. paracasei* AO356 with taurodeoxycholic acid (TDCA), which is one of the bile salts, and the result was negative (**Supplementary Figure 2B**). To verify the safety of *L. paracasei* AO356 for virulence or toxin-related propertes, we performed an LDH assay (**Supplementary Figure 2C**). The cytotoxicity of caco-2 cells co-cultured with *E. coli* was 95–100%, whereas the cytotoxicity of caco-2 cells co-cultured with *L. paracasei* AO356 was 17–22%. The cytotoxicity of *L. paracasei* AO356 was similar to that of the low control (21%) treated only media. In measurement of D-lactate, *L. paracasei* AO356 produced 2.18 g/L (94.30%) of L-lactate and 0.14 g/L (5.70%) of D-lactate. The level of D-lactate production was lower than that of *Lactobacillus rhamnosus* GG which is one of the most commonly used probiotic strain (**Supplementary Figure 2E**). These results suggested that *L. paracasei* AO356 can be considered a safe strain and used as a healthy functional food ingredient.

## 4 Discussion

In this study, the *L. paracasei* AO356 intervention prevented weight gain and fat accumulation without changes in energy intake. The *L. paracasei* AO356 intervention also modulated the overall composition of gut microbiota and changed the gut microbiota at the genus level. Additionally, the *L. paracasei* AO356 intervention also modulated the predicted functional metagenomic profiles and changed the pathways correlated with weight gain and fat accumulation. Furthermore, we have secured the safety of *L. paracasei* AO356 for antibiotics resistance, hemolysis activity, bile salt hydrolase activity, toxicity, and D-lactate production. These results indicate that *L. paracasei* AO356 has an anti-obesity effect, can modulate gut microbiota, and is guaranteed to be safe as a probiotic and healthy functional food.

Our previous study investigated the effects of *L. paracasei* AO356 on an obesity mice model concerning aspects of regulating adipogenesis and thermogenesis (9). The study revealed that the anti-obesity effect of *L. paracasei* AO356 may be associated with the downregulation of adipogenesis transcription factor and lipid metabolism-related genes, such as *Srebp1c*, *Pparγ*, *Fas*, *C/ebpα*, and *Fabp4* and upregulation of thermogenesis-related genes, such as *Ucp1*, *Cpt1*, *Pgc1α*, *Cidea*, and *Prdm16* in epididymal and subcutaneous fat pads. The study also compared the anti-obesity effects of *L. paracasei* AO356 at two concentrations of 1× 10^7^ CFU/head or 1 × 10^8^ CFU/head. There was no significant difference in the anti-obesity effect of *L. paracasei* between the two concentrations, which suggested that *L. paracasei* AO356 showed anti-obesity activity at doses > 10^7^ CFU/head. However, the previous study did not explore the effect of *L. paracasei* AO356 on gut microbiota. Therefore, in this study, we investigated the influence of *L. paracasei* AO356 and its anti-obesity effects on the composition of gut microbiota and predicted metagenomic profiles at an intermediate concentration of 5 × 10^7^ CFU/head.

In this study, *Prevotella* and *Bacteroides* were the most characteristic genera in the ND and HFD groups, respectively. The association of *Prevotella* and *Bacteroides* with diet and obesity has been reported. Industrialized populations consuming Western diets, low in fiber and high in fat and processed sugar, have a gut microbiota dominated by *Bacteroidaceae* (whose representative genus is *Bacteroides*), whereas traditional populations have a gut microbiota dominated by *Prevotellaceae* (whose representative genus is *Prevotella*) (30). Individuals whose guts are enriched with *Bacteroides* have a higher incidence of diseases associated with a Western diet than individuals whose guts are enriched with *Prevotella* (31). *Bacteroides* were prominent among obese individuals and positively correlated with BMI (32). Additionally, an abundance of *Prevotella* and *Bacteroides* are associated with anti-obesity susceptibility. Individuals with high *Prevotella*/*Bacteroides* ratios are more susceptible to loss of body weight and body fat after a high-fiber diet (33, 34). Anti-obesity probiotics, including complexes of *Lactobacillus plantarum* CBT LP3 and *Bifidobacterium breve* CBT BR3, were not effective in individuals whose guts were enriched with *Bacteroides* (35). The rate of decrease in BMI in individuals whose guts were enriched with *Prevotella* was higher than in individuals whose guts were enriched with *Bacteroides* after 3 weeks of a calorie-restriction diet (36). Our results showed that the *L. paracasei* AO356 intervention restored the relative abundance of *Bacteroides* which was increased by the HFD feeding. Our results, in combination with previous literature, suggest that decreased relative abundance of *Bacteroides* after *L. paracasei* AO356 intervention may have contributed to increased susceptibility to body weight and body fat loss in HFD-induced obese mice.

We also discovered that the *L. paracasei* AO356 intervention significantly increased the relative abundance of *Oscillospira*. *Oscillospira* showed a negative correlation with obesity-related markers and a negative correlation with metabolic pathways such as unsaturated fatty acid biosynthesis, fatty acid biosynthesis, and butanoate metabolism. Several clinical studies found that individuals with lower BMIs had guts that were more enriched *Oscillospira* compared to those with a higher BMI and that *Oscillospira* was negatively correlated with BMI (37–41). Recently, *Oscillospira* was proposed as a candidate for next-generation probiotics (42). However, the mechanism by which *Oscillospira* acts on obesity is still unclear. Chen et al. suggested that the glycan degradation properties of *Oscillospira* were a possible mechanism for the reason why *Oscillospira* is related to leanness (43). Hosts need to consume energy to regenerate the degraded glycan that constitutes gut mucins degraded by *Oscillospira*. In our study, the anti-obesity effect of *L. paracasei* AO356 and enriched *Oscillospira* was correlated with the biosynthesis of unsaturated fatty acids. Unsaturated fatty acids are a type of fatty acid that have one or more double bonds in their chemical structure. Omega-3 fatty acids are well known unsaturated fatty acids and have health beneficial properties such as anti-inflammatory effects and hypotriglyceridemia. They also improve body composition and obesity-related metabolic changes including lipid metabolism (44, 45). Conjugated linoleic acid (CLA), one of the unsaturated fatty acids, has positive health effects on body composition and blood lipid concentrations (46). Diets rich in unsaturated fatty acids improve blood glucose control (47). Given that *Oscillospira*, obesity-related markers and metabolic pathways are correlated, and in light of previous literature, further research should be conducted to determine whether the anti-obesity effect of *L. paracasei* AO356 is caused by gut microbial changes.

Butanoate, also known as butyrate, and propanoate, also known as propionate, are short-chain fatty acids that are produced by bacteria in the gut during the fermentation of dietary fiber (48). Butanoate metabolism involves the breakdown of butanoate into acetyl-CoA, which can then be used as a source of energy by the body. Propanoate metabolism involves the conversion of succinate to methylmalonyl-CoA, which can then enter the citric acid cycle to produce energy. Butanoate and propanoate metabolism are important for maintaining gut health by reducing inflammation and improving gut function and energy metabolism (49–51). In this study, we found a positive correlation between enriched carbohydrate metabolism-related pathways (butanoate metabolism and propanoate metabolism) and *Oscillospira* after the *L. paracasei* AO356 intervention. Similar to our result, a study showed that *Pleurotus ostreatus* had a preventive effect on obesity and beneficially modulated gut microbiota, including increased relative abundance of *Oscillospira* and the butanoate and propanoate metabolism pathway (52). Other studies also showed that propanoate metabolism was enriched after *Lactobacillus plantarum* HNU082 intervention in an HFD-induced hyperlipidemia rat model. Propanoate metabolism was also linked to improvements in the gut microbiota and the prevention of hyperlipidemia (53). Our results showed that an enriched propanoate metabolism pathway after *L. paracasei* AO356 intervention was negatively correlated with serum triglycerides. Further studies to determine whether *Oscillospira* has an anti-obesity effect via the butanoate and propanoate metabolism pathways will help elucidate the mechanism of the anti-obesity effect of *L. paracasei* AO356.

Additionally, we found that the isoflavonoid biosynthesis pathway was enriched after the *L. paracasei* AO356 intervention. Isoflavonoids are a class of phytoestrogens that are found in soybeans and other legumes and have been associated with various health benefits, including anti-inflammatory and anti-cancer effects (54). Recent research has also highlighted the potential role of isoflavonoids in the regulation of body weight and metabolism. For example, a meta-analysis study with 17 trials found that isoflavonoids tended to decrease body mass index (55). An animal study found that isoflavonoids decreased body weight and adipose tissue weight (56). The mechanism of action of isoflavonoids on obesity was found to be the inhibition of fat production and increased fatty acid β-oxidation, which leads to reduced body fat (57, 58). Another suggested mechanism is that isoflavonoids interact with intracellular estrogen receptors, which results in reductions in the accumulation of lipids and the distribution of adipose tissue (59). Additionally, the isoflavonoid biosynthesis pathway includes the metabolism of isoflavonoids, such as daidzein and genistein, into more bioactive compounds, such as equol, which has been shown to have anti-obesity effects (60–62). Conversion of isoflavonoids into bioactive compounds mediated by gut microbiota is also proposed as one of the mechanisms of action of isoflavonoids on obesity. Overall, our findings suggest that the anti-obesity effects of *L. paracasei* AO356 may be mediated, at least in part, by changes in gut microbiota function, including the enrichment of the isoflavonoid biosynthesis pathway. However, further studies are needed to elucidate the role of the isoflavonoid biosynthesis pathway in the anti-obesity effect of *L. paracasei* AO356.

Recently, as side effects from taking probiotics or migration of antibiotic resistance genes through probiotic strains have become a problem, each country presented guidelines for the safety of probiotics (19). In previous studies, the safety of *L. paracasei* AO356 was evaluated before the presentation of the guideline from the Korea Ministry of Food and Drug Safety (MFDS) (24). To evaluate the safety, the study conducted including antibiotics resistance tests, a hemolytic test, and enzyme (gelatinase and β-glucuronidase) activity tests in previous study. In 2021, MFDS presented guidelines that included the evaluation of antibiotics resistance, hemolysis activity, bile salt hydrolase (BSH) activity, toxicity, and D-lactate production (19). Therefore, we additionally evaluated the safety of *L. paracasei* AO356 according to the MFDS guideline. Some strains of lactic acid bacteria (LAB) isolated from fermented foods have been reported to have resistance to antibiotics including erythromycin and tetracycline (63, 64). In addition, LAB play role as a storage for antibiotic-resistant genes and can transfer them to other bacteria (65). Antibiotic resistance is an important for health issue because it can increase morbidity and mortality in patients. For this reason, there is an increasing concern in antibiotic-resistant bacteria in the field of foods and medicine, and antibiotic resistance must be confirmed for the safety of probiotics. In our study, the assessment of antibiotic resistance with E-test demonstrated that *L. paparacasei* AO356 is a safe strain due to its sensitivity accorded with the EFSA-presented antibiotics guidance for *L. paracasei* species. Hemolysis, which is virulence factor, is a lysis of red blood cells by hemolysins secreted by bacteria. In particular, the absence of hemolytic activity is a critical factor in probiotics selection because beta-hemolysis could lead hemolytic anemia by destroying red blood cells (66). Also, hemolysis affects the innate and adaptive immune system and people with chronic inflammatory diseases are particularly vulnerable to infection (67). As with the results that most LAB strains had no hemolytic activity, it was observed that *L. paracasei* AO356 also did not have a hemolytic activity (68). BSH produced by intestinal bacteria is involved in the first steps of bile acid transformation in catalyzing hydrolysis of conjugated bile slats (69). The BSH activity has been reported to be related to functional activity such as serum cholesterol reduction and anti-obesity activity (70); However, there are some negative concerns regarding BSH activity, such as the potential production of cytotoxic and carcinogenic secondary bile salts (71). The enriched deconjugated bile acids by BSH can lead to diarrhea, promoted intestinal inflammation, cholestasis (72). Furthermore, deconjugated bile acids are subsequently transformed into secondary bile acids such as deoxycholic acid (DCA) and lithocholic acid (LCA), by 7-α-dehydroxylase (69). These secondary bile acids have been reported to be cytotoxic and co-carconogenic *in vitro*, and related to colorectal cancer (71, 73). In this study, we confirmed that *L. paracasei* AO356 have no BSH activity and it indicated that *L. paracasei* AO356 with minimal stability concerns in toxic substances produced by BSH. Other toxin and virulence properties were confirmed through the cytotoxicity of the *L. paracasei* AO356 for Caco-2 cell line. The Cytotoxicity was verified by measurement of LDH concentration released from damaged cells (74). *E.coli* (ACTC1682) is known to be toxic and induced cell damage similar to treated cell lysis buffer, but our finding demonstrated athat *L. paracasei* AO356 showed no toxicity to Caco-2 cell line. Finally, we evaluated the capacity to produce D-lactate of *L. paracasei* AO356. Some *Lactobacillus* spp. can produce D-lactate as well as L-lactate. In human, while L-lactate is rapidly metabolized to pyruvate, D-lactate is restrictively metabolized by D-α-hydroxy acid dehydrogenase, an enzyme produced by intestinal microbes (75). The excessive accumulation of D-lacatate in blood (≥3 mmol/L in healthy people or 2.5-3.0 mmol/L in patient with short bowel syndrome) can lead to D-lactic acidosis accompanied by various neurologic manifestations (75). Particularly, it could be severe problems in infants an in patients with short bowel syndrome. Thus, the FAO/WHO requires information about D-lactate production to assess the safety of probiotics, and recommended that the product label should specify the information about strains that produce D-lacate (76, 77). In this study, we confirmed that *L. paracasei* AO356 mainly produced L-lactate and rarely produced D-lactate, which is similar to *L. rhamnosus GG*, commercial probiotics. *L. paracasei* AO356 has secured safety for all indicators presented by the guideline. These findings indicated that *L. paracasei* AO356 meets the safety criteria for probiotics recommended by EFSA and MFDA, thereby suggesting its potential as safe probiotics.

In summary, we showed that the *L. paracasei* AO356 intervention attenuated the development of HFD-induced obesity in mice. The *L. paracasei* AO356 intervention also modulated gut microbiota, which may be associated with anti-obesity related markers, biosynthesis of other secondary metabolites-related pathways (isoflavonoid biosynthesis), carbohydrate metabolism-related pathways (butanoate metabolism, propanoate metabolism, and pentose phosphate metabolism) and lipid metabolism-related pathways (biosynthesis of unsaturated fatty acids, fatty acid metabolism, and ether lipid metabolism). *L. paracasei* AO356 with anti-obesity effects can modulate gut microbiota, however more accurate mechanisms should be studied to evaluate whether the anti-obesity effect is caused by changes in gut microbiota induced by *L. paracasei* AO356. Moreover, *L. paracasei* AO356 conformed to the safety assessment guides presented by the MFDS and is therefore suitable for the development of probiotics as a healthy functional food and especially as an alternative therapeutic target for obesity prevention and treatment.

## Data availability statement

The datasets presented in this study can be found in online repositories. The names of the repository/repositories and accession number(s) can be found below: https://www.ddbj.nig.ac.jp/, DRA006554.

## Author contributions

E-JS and E-SL analyzed and interpreted the data, and drafted the manuscript. YK acquired and analyzed the data, D-US and J-EE acquired the data, and HS conceptualized and supervised the research. S-YL and Y-DM conceptualized and supervised the research and performed a critical revision of the manuscript. All authors contributed to the article and approved the submitted version.
