# Supplementary material for: Gut microbial change after administration of Lacticaseibacillus paracasei AO356 is associated with anti-obesity in a mouse model
Source: Front Endocrinol (Lausanne). 2023 Aug 29;14:1224636. doi: 10.3389/fendo.2023.1224636 (PMC10496115; doi:10.3389/fendo.2023.1224636)
Supplement: Supplementary file 1 [file DataSheet_1.docx]

Supplementary Material

# Supplementary Tables

**Supplementary Table 1.** LEfSe results of predicted functional metagenome by PICRUSt

| **Hierarchical Level 1** | **Hierarchical Level 2** | **Hierarchical Level 3** | **Group** | **LDA** |
| --- | --- | --- | --- | --- |
| Environmental Information Processing | Membrane Transport | Secretion system | HFD | 3.20 |
| Environmental Information Processing | Membrane Transport | Transporters | HFD | 4.12 |
| Environmental Information Processing | Signal Transduction | Two-component system | HFD | 3.58 |
| Metabolism | Enzyme Families | Protein kinases | HFD | 2.76 |
| Metabolism | Lipid Metabolism | Glycerolipid metabolism | HFD | 2.35 |
| Metabolism | Metabolism of Cofactors and Vitamins | Porphyrin and chlorophyll metabolism | HFD | 3.29 |
| Environmental Information Processing | Membrane Transport | ABC transporters | HFD+AO356 | 3.83 |
| Environmental Information Processing | Membrane Transport | Phosphotransferase system (PTS) | HFD+AO356 | 3.11 |
| Metabolism | Amino Acid Metabolism | Lysine degradation | HFD+AO356 | 2.09 |
| Metabolism | Amino Acid Metabolism | Phenylalanine metabolism | HFD+AO356 | 2.02 |
| Metabolism | Biosynthesis of Other Secondary Metabolites | Isoflavonoid biosynthesis | HFD+AO356 | 3.08 |
| Metabolism | Carbohydrate Metabolism | Butanoate metabolism | HFD+AO356 | 2.43 |
| Metabolism | Carbohydrate Metabolism | Glyoxylate and dicarboxylate metabolism | HFD+AO356 | 2.18 |
| Metabolism | Carbohydrate Metabolism | Pentose phosphate pathway | HFD+AO356 | 2.64 |
| Metabolism | Carbohydrate Metabolism | Propanoate metabolism | HFD+AO356 | 2.64 |
| Metabolism | Lipid Metabolism | Biosynthesis of unsaturated fatty acids | HFD+AO356 | 2.10 |
| Metabolism | Lipid Metabolism | Ether lipid metabolism | HFD+AO356 | 2.54 |
| Metabolism | Lipid Metabolism | Fatty acid biosynthesis | HFD+AO356 | 2.17 |
| Metabolism | Lipid Metabolism | Fatty acid metabolism | HFD+AO356 | 2.36 |
| Metabolism | Lipid Metabolism | Steroid biosynthesis | HFD+AO356 | 2.69 |
| Metabolism | Lipid Metabolism | Synthesis and degradation of ketone bodies | HFD+AO356 | 2.19 |
| Metabolism | Metabolism of Terpenoids and Polyketides | Biosynthesis of ansamycins | HFD+AO356 | 2.21 |
| Metabolism | Metabolism of Terpenoids and Polyketides | Tetracycline biosynthesis | HFD+AO356 | 2.40 |
| Metabolism | Xenobiotics Biodegradation and Metabolism | Benzoate degradation | HFD+AO356 | 2.50 |
| Metabolism | Xenobiotics Biodegradation and Metabolism | Chloroalkane and chloroalkene degradation | HFD+AO356 | 2.51 |
| Metabolism | Xenobiotics Biodegradation and Metabolism | Dioxin degradation | HFD+AO356 | 2.23 |
| Metabolism | Xenobiotics Biodegradation and Metabolism | Drug metabolism - cytochrome P450 | HFD+AO356 | 2.04 |
| Metabolism | Xenobiotics Biodegradation and Metabolism | Metabolism of xenobiotics by cytochrome P450 | HFD+AO356 | 2.04 |
| Metabolism | Xenobiotics Biodegradation and Metabolism | Nitrotoluene degradation | HFD+AO356 | 2.57 |
| Metabolism | Xenobiotics Biodegradation and Metabolism | Xylene degradation | HFD+AO356 | 2.14 |
| Organismal Systems | Environmental Adaptation | Plant-pathogen interaction | HFD+AO356 | 2.39 |
| Environmental Information Processing | Membrane Transport | Bacterial secretion system | ND | 2.55 |
| Environmental Information Processing | Signaling Molecules and Interaction | Cellular antigens | ND | 2.36 |
| Metabolism | Amino Acid Metabolism | Alanine, aspartate and glutamate metabolism | ND | 3.01 |
| Metabolism | Amino Acid Metabolism | Amino acid related enzymes | ND | 2.91 |
| Metabolism | Amino Acid Metabolism | Arginine and proline metabolism | ND | 2.85 |
| Metabolism | Amino Acid Metabolism | Cysteine and methionine metabolism | ND | 2.70 |
| Metabolism | Amino Acid Metabolism | Glycine, serine and threonine metabolism | ND | 2.99 |
| Metabolism | Amino Acid Metabolism | Histidine metabolism | ND | 2.80 |
| Metabolism | Amino Acid Metabolism | Lysine biosynthesis | ND | 2.75 |
| Metabolism | Amino Acid Metabolism | Phenylalanine, tyrosine and tryptophan biosynthesis | ND | 2.85 |
| Metabolism | Amino Acid Metabolism | Valine, leucine and isoleucine biosynthesis | ND | 2.55 |
| Metabolism | Biosynthesis of Other Secondary Metabolites | beta-Lactam resistance | ND | 2.11 |
| Metabolism | Biosynthesis of Other Secondary Metabolites | Butirosin and neomycin biosynthesis | ND | 2.15 |
| Metabolism | Biosynthesis of Other Secondary Metabolites | Novobiocin biosynthesis | ND | 2.10 |
| Metabolism | Biosynthesis of Other Secondary Metabolites | Penicillin and cephalosporin biosynthesis | ND | 2.09 |
| Metabolism | Biosynthesis of Other Secondary Metabolites | Phenylpropanoid biosynthesis | ND | 2.59 |
| Metabolism | Biosynthesis of Other Secondary Metabolites | Streptomycin biosynthesis | ND | 2.78 |
| Metabolism | Carbohydrate Metabolism | Amino sugar and nucleotide sugar metabolism | ND | 2.46 |
| Metabolism | Carbohydrate Metabolism | Citrate cycle (TCA cycle) | ND | 3.05 |
| Metabolism | Carbohydrate Metabolism | Galactose metabolism | ND | 2.95 |
| Metabolism | Carbohydrate Metabolism | Pentose and glucuronate interconversions | ND | 2.44 |
| Metabolism | Carbohydrate Metabolism | Starch and sucrose metabolism | ND | 2.84 |
| Metabolism | Energy Metabolism | Carbon fixation in photosynthetic organisms | ND | 2.49 |
| Metabolism | Energy Metabolism | Carbon fixation pathways in prokaryotes | ND | 2.98 |
| Metabolism | Energy Metabolism | Nitrogen metabolism | ND | 2.23 |
| Metabolism | Energy Metabolism | Oxidative phosphorylation | ND | 3.26 |
| Metabolism | Energy Metabolism | Photosynthesis | ND | 2.47 |
| Metabolism | Energy Metabolism | Photosynthesis proteins | ND | 2.38 |
| Metabolism | Enzyme Families | Peptidases | ND | 3.13 |
| Metabolism | Glycan Biosynthesis and Metabolism | Glycosaminoglycan degradation | ND | 2.88 |
| Metabolism | Glycan Biosynthesis and Metabolism | Glycosphingolipid biosynthesis - ganglio series | ND | 2.82 |
| Metabolism | Glycan Biosynthesis and Metabolism | Glycosphingolipid biosynthesis - globo series | ND | 2.91 |
| Metabolism | Glycan Biosynthesis and Metabolism | Glycosyltransferases | ND | 2.39 |
| Metabolism | Glycan Biosynthesis and Metabolism | Lipopolysaccharide biosynthesis | ND | 3.04 |
| Metabolism | Glycan Biosynthesis and Metabolism | Lipopolysaccharide biosynthesis proteins | ND | 3.07 |
| Metabolism | Glycan Biosynthesis and Metabolism | N-Glycan biosynthesis | ND | 2.04 |
| Metabolism | Glycan Biosynthesis and Metabolism | Other glycan degradation | ND | 3.22 |
| Metabolism | Glycan Biosynthesis and Metabolism | Peptidoglycan biosynthesis | ND | 2.55 |
| Metabolism | Lipid Metabolism | Lipid biosynthesis proteins | ND | 2.32 |
| Metabolism | Lipid Metabolism | Sphingolipid metabolism | ND | 2.92 |
| Metabolism | Metabolism of Cofactors and Vitamins | Biotin metabolism | ND | 2.46 |
| Metabolism | Metabolism of Cofactors and Vitamins | Folate biosynthesis | ND | 2.92 |
| Metabolism | Metabolism of Cofactors and Vitamins | Lipoic acid metabolism | ND | 2.27 |
| Metabolism | Metabolism of Cofactors and Vitamins | Nicotinate and nicotinamide metabolism | ND | 2.77 |
| Metabolism | Metabolism of Cofactors and Vitamins | One carbon pool by folate | ND | 2.98 |
| Metabolism | Metabolism of Cofactors and Vitamins | Pantothenate and CoA biosynthesis | ND | 2.77 |
| Metabolism | Metabolism of Cofactors and Vitamins | Riboflavin metabolism | ND | 2.41 |
| Metabolism | Metabolism of Cofactors and Vitamins | Thiamine metabolism | ND | 2.11 |
| Metabolism | Metabolism of Cofactors and Vitamins | Ubiquinone and other terpenoid-quinone biosynthesis | ND | 2.91 |
| Metabolism | Metabolism of Cofactors and Vitamins | Vitamin B6 metabolism | ND | 2.56 |
| Metabolism | Metabolism of Other Amino Acids | beta-Alanine metabolism | ND | 2.67 |
| Metabolism | Metabolism of Other Amino Acids | Cyanoamino acid metabolism | ND | 2.74 |
| Metabolism | Metabolism of Other Amino Acids | D-Glutamine and D-glutamate metabolism | ND | 2.37 |
| Metabolism | Metabolism of Other Amino Acids | Glutathione metabolism | ND | 2.12 |
| Metabolism | Metabolism of Other Amino Acids | Taurine and hypotaurine metabolism | ND | 2.03 |
| Metabolism | Metabolism of Terpenoids and Polyketides | Biosynthesis of vancomycin group antibiotics | ND | 2.18 |
| Metabolism | Metabolism of Terpenoids and Polyketides | Geraniol degradation | ND | 2.01 |
| Metabolism | Metabolism of Terpenoids and Polyketides | Polyketide sugar unit biosynthesis | ND | 2.62 |
| Metabolism | Metabolism of Terpenoids and Polyketides | Prenyltransferases | ND | 2.72 |
| Metabolism | Metabolism of Terpenoids and Polyketides | Terpenoid backbone biosynthesis | ND | 2.69 |
| Metabolism | Nucleotide Metabolism | Purine metabolism | ND | 3.15 |
| Metabolism | Nucleotide Metabolism | Pyrimidine metabolism | ND | 3.24 |
| Metabolism | Xenobiotics Biodegradation and Metabolism | 1,1,1-Trichloro-2,2-bis(4-chlorophenyl)ethane (DDT) degradation | ND | 3.05 |
| Metabolism | Xenobiotics Biodegradation and Metabolism | Drug metabolism - other enzymes | ND | 2.62 |
| Metabolism | Xenobiotics Biodegradation and Metabolism | Toluene degradation | ND | 2.42 |
| Organismal Systems | Digestive System | Protein digestion and absorption | ND | 2.26 |
| Organismal Systems | Endocrine System | Adipocytokine signaling pathway | ND | 2.29 |

ND, normal diet mice; HFD, HFD mice; HFD+AO356, HFD mice with *Lacticaseibacillus paracasei* AO356 intervention; LDA, linear discriminant analysis score (log 10)

**Supplementary Table 2.** Minimum inhibitory concentration (MIC) of antibiotics of *Lacticaseibacillus paracasei* AO356

| Antibiotics | MIC Cut-off value of EFSA^1)^ for *Lactobacillus paracasei*  (mg/mL) | MIC (mg/mL)  of AO356 |
| --- | --- | --- |
| Ampicillin | 4 | 0.25 ± 0.00 |
| Chloramphenicol | 4 | 0.75 ± 0.00 |
| Clindamycin | 1 | <0.016 ± 0.00 |
| Erythromycin | 1 | 0.064 ± 0.00 |
| Gentamicin | 32 | 16 ± 0.00 |
| Kanamycin | 64 | 8 ± 0.00 |
| Streptomycin | 64 | 32 ± 0.00 |
| Tetracycline | 4 | 0.125 ± 0.00 |
| Vancomycin | n.r.^2)^ | 2 ± 0.00 |

The Minimum Inhibitory Concentration (MIC) values of *Lacticaseibacillus paracasei* AO356 for ampicillin, chloramphenicol, clindamycin, erythromycin, gentamicin, kanamycin, streptomycin, tetracycline, and vancomycin. ^1)^ ESFA, European Food Safety Authority ^2)^ n.r. not required

# Supplementary Figures

**
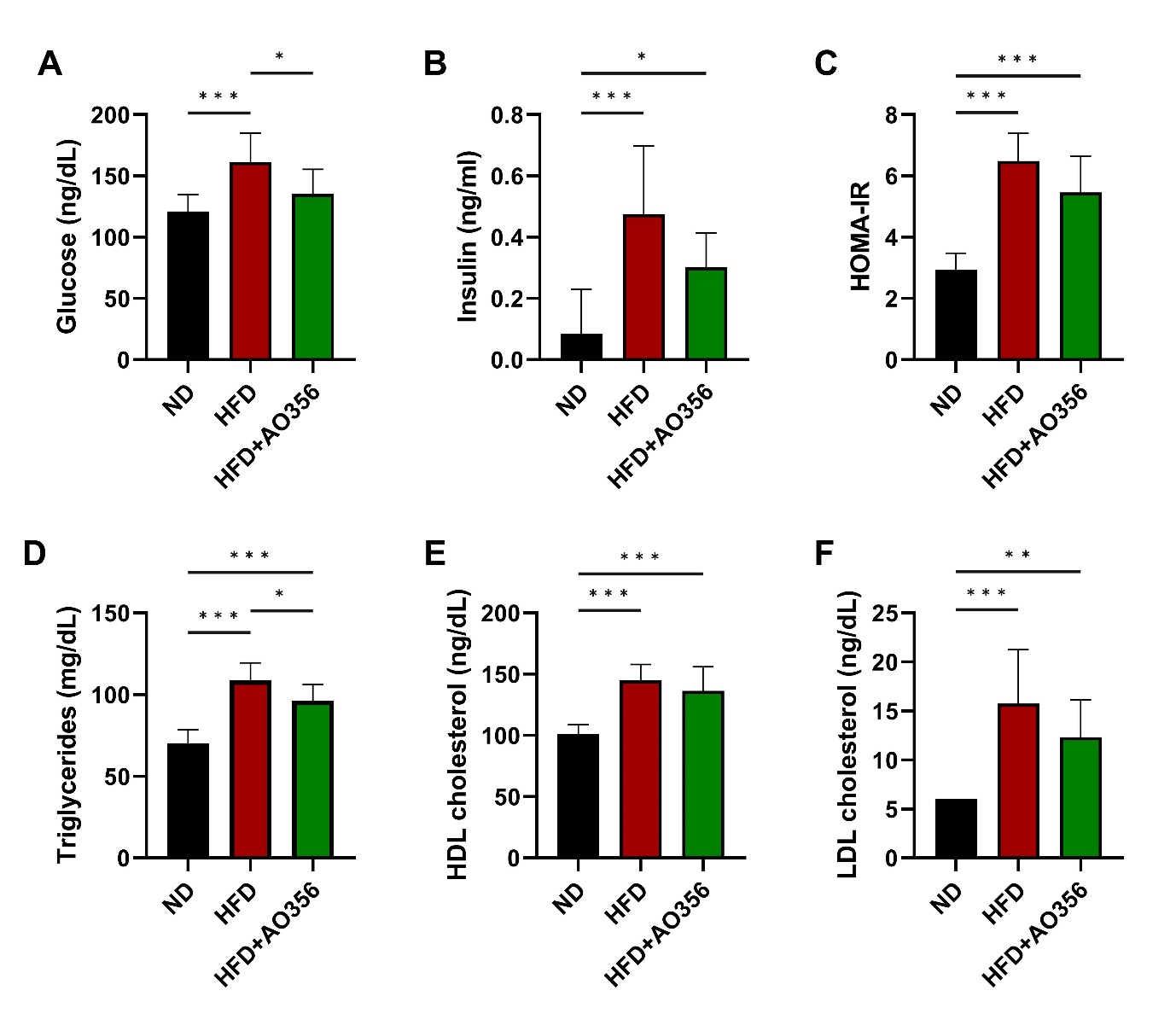
**

**Supplementary Figure 1. Effect of *L. paracasei* AO356 on insulin resistance and serum lipid profile in a high-fat diet (HFD)-induced obese model after intervention for 10 weeks.** (A) Serum glucose (B) Serum insulin (C) HOMA-IR (D) Serum triglycerides (E) Serum HDL cholesterol (F) Serum LDL cholesterol. ND, normal diet mice; HFD, HFD mice; HFD+AO356, HFD mice with *Lacticaseibacillus paracasei* AO356 intervention. Statistical significance was assessed using one-way ANOVA and Tukey’s multiple comparison test. *P <0.05, **P <0.01, ***P <0.001.


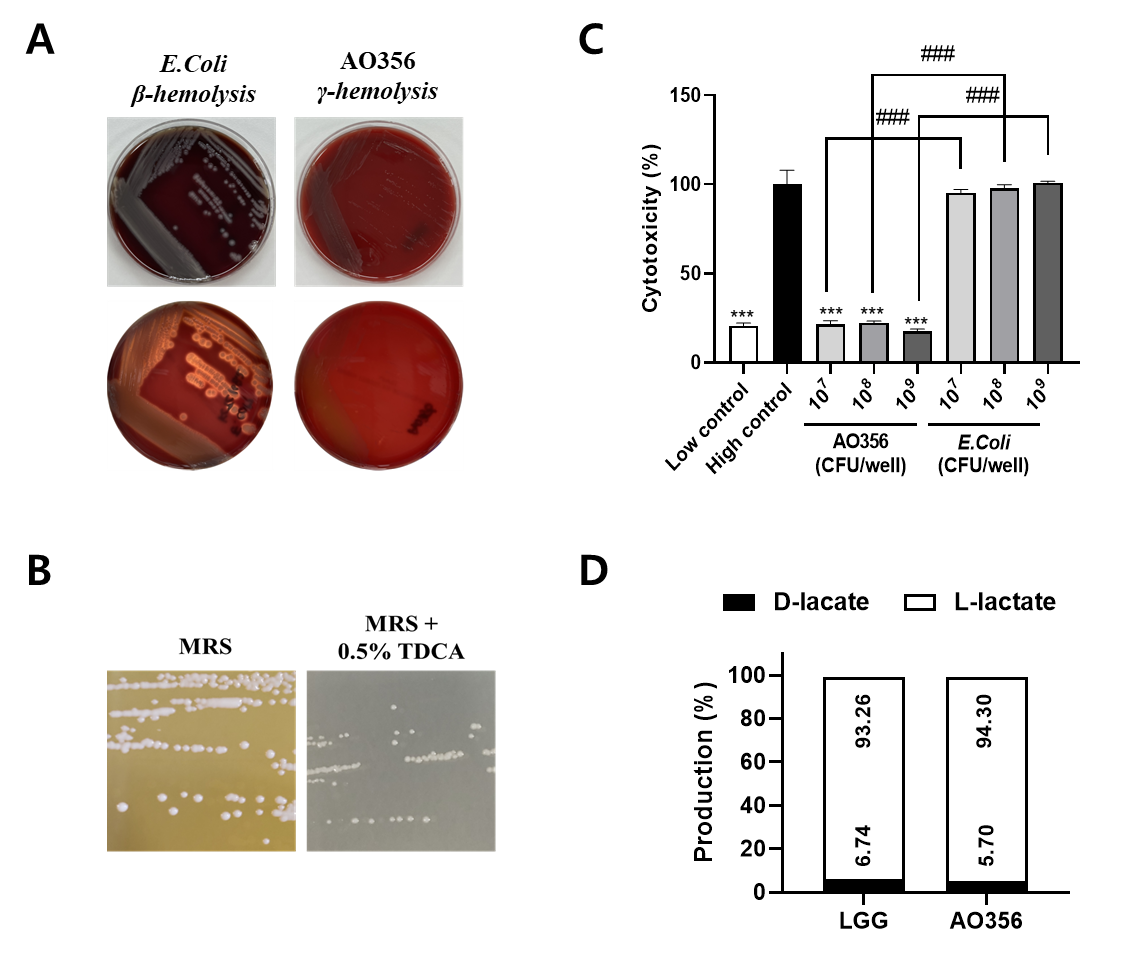


**Supplementary Figure 2. The safety assessment of *Lacticaseibacillus paracasei* AO356. (A)** Hemolysis activity test was conducted on sheep blood agar plate. *Escherichia coli*, known for its toxicity, was used as a positive strain. **(B)** Bile salt hydrolase activity test. **(C)** The cytotoxicity on the Caco-2 cell line was determined using LDH assay. The non-treated culture medium was used as a low control, the cell lysis buffer was used as a high control, and *Escherichia coli* (ACTC1682), known to be toxic, was used as a positive control. **(D)** D-lactate production test. *Lactobacillus rhamnosus*, known for its safety, was used as a positive strain. *<0.05, **<0.01, ***<0.001 by ANOVA with Dunnett test. The asterisk indicates a statistically significant difference with high control. The # symbol indicates a statistically significant difference between the AO356 and *Escherichia coli*.
